# A contemporary review of adult bladder trauma

**DOI:** 10.5249/jivr.v11i2.1069

**Published:** 2019-07

**Authors:** Yashmi Mahat, Joon Yau Leong, Paul H. Chung

**Affiliations:** ^*a*^Department of Urology, Sidney Kimmel Medical College, Thomas Jefferson University, Philadelphia, PA, USA.

**Keywords:** Bladder, Trauma, Extraperitoneal, Intraperitoneal, Iatrogenic

## Abstract

Injuries to the bladder are infrequent and commonly result from blunt, penetrating, or iatrogenic trauma. Bladder injuries may be missed as they often present concomitantly with other abdominal and pelvic injuries; however, early detection and treatment are essential as morbidity and mortality may be significant. Gross hematuria, especially in the setting of pelvic fractures, may be indicative of a bladder injury which can be confirmed with cystography. Extraperitoneal injuries are commonly managed conservatively with catheter drainage while intraperitoneal ruptures traditionally required surgical exploration and closure. Presented is a contemporary review which encapsulates the etiology, presentation, assessment, and management of bladder injuries.

## Introduction

Injuries to the bladder occur in up to 10% of abdominal trauma and may be associated with significant morbidity and mortality (10-22%).^1,2^ Bladder injuries more specifically may result from blunt or penetrating trauma and iatrogenic injury during surgery. Cystography may be performed to diagnose the presence and grade of bladder injury which will subsequently guide whether conservatively management or surgical intervention is required. This review encapsulates the etiology, presentation, assessment, and management of bladder injuries.

**Etiology**

The bladder is an extraperitoneal organ that is protected by the pubic bone. In males, it sits superior and anterior to the prostate, while in females it lies anterior to the uterus. Superior and posterior to the bladder is the peritoneum, a membranous layer that delineates the intraabdominal cavity. Bladder injury can, therefore, be subdivided into extraperitoneal (EP), intraperitoneal (IP), or combined EP and IP; accounting for 63%, 32%, and 4% of cases, respectively.^3^ Another form of bladder injury, the interstitial subtype, is uncommon and is an incomplete disruption of the bladder wall without extravasation of urine. Ratios of EP and IP injuries may vary based on geography and mechanism of injury. ^2,4^ In one study from South Africa, IP injuries were more common, representing 60% of bladder injuries, while EP injuries represented 22%. This may be due to penetrating injuries being more common (65%) than blunt trauma (22%) at the respective trauma centers.^4^ Another institution evaluated their series of gunshot wounds to the lower urinary tract and identified that 72% of patients suffered from bladder injury and 80% had a concurrent gastrointestinal injury, further demonstrating that penetrating injuries are at risk for IP injury. ^5^

Blunt trauma accounts for 60-85%, while penetrating trauma accounts for 15-51% of bladder injuries.^2,3,5,6,7^ Blunt abdominal injuries are most notably due to motor-vehicle accidents while penetrating injuries often result from stab or gunshot wounds (GSW). GSWs are responsible for the majority of penetrating bladder trauma compared to stab wounds (80% versus 20%) in the United States.^8^ GSWs are high-velocity injuries that may follow an unpredictable path and result in a blast effect which inflicts more significant damage to the surrounding tissue. Stab wounds follow a more predictable path limited to the immediate trajectory of the object. 

Several mechanisms of blunt bladder injury have been proposed. Direct force to the abdomen can cause a “burst” rupture of the dome, the weakest part of the bladder. A filled bladder is more susceptible to rupture because the dome rises into the abdominal cavity, eliminating protection afforded by the bony pelvis and pelvic organs. This leads to an IP bladder injury and urine extravasation into the peritoneal cavity, which carries a risk for peritonitis, chemical ileus, sepsis, and even death. Although concomitant pelvic injuries are not uncommon in IP injury, up to 25% do not have concomitant pelvic injuries.^9^

Bladder injuries are often associated with concomitant pelvic fractures in 85-100% of cases.^8-10^ These injuries may cause an EP rupture, where urine may leak into the perivesicular space surrounding the bladder but does not enter the intraperitoneal cavity. Pelvic ring disruptions may create a shear force disrupting ligaments holding the bladder wall to the base of the pelvis or a counter-coup force that results in a burst injury opposite to the site of the pelvic fracture. In 65% of cases, the injury to the bladder is opposite the area of fracture.^11^ Moreover, bony fragments from a pelvic fracture may also directly lacerate the bladder surface.

Other associated injuries in bladder trauma include long bone fractures, central nervous system, and thoracic injuries, as well as other intra-abdominal injuries.^8,10^ The high mortality seen with bladder injuries stems from other associated injuries rather than the bladder injury itself.^12^ Neighboring organs to the bladder are also at risk for injury. Penetrating bladder trauma can be associated with concomitant rectal injuries in up to 38% of patients, which may lead to greater morbidity through contamination of bowel contents and possible sepsis.^2^

Isolated bladder injuries are rare, with most being secondary to iatrogenic causes.^1^ Iatrogenic bladder injuries are highest in gynecologic and urologic surgeries, given the proximity of structures in the pelvis, but may also occur with general and orthopedic surgeries. Procedures associated with the highest incidence of bladder injuries include vaginal hysterectomies (0.4-6.3%), urethral or retropubic slings (6-50%), and transurethral resection of the bladder (3.5-58%).^13^

The American Association of Surgery for Trauma (AAST) developed the Organ Injury Scale to provide a common language to facilitate clinical decision making and research. It is based on the degree of anatomical disruption with Grade I being mild to Grade V being lethal. Bladder injury is graded as a contusion or partial laceration (Grade I) to complete laceration (Grades II-V) (Table 1). ^14^ Grade I injuries, contusions of the bladder wall and partial thickness lacerations, can lead to self-limiting intramural hematoma formation.^12^ These minor injuries are the most common injuries and represent a third of all cases of bladder injury. ^11^ EP injuries are Grade II ( <2 cm) or Grade III (≥2 cm). IP injuries are Grade III ( <2 cm) or Grade IV (≥2 cm). Bladder injuries may extend down to the bladder neck and involve the ureteral orifices or trigone (Grade V). ^15^ Detecting these injuries is essential because an unrecognized injury to the bladder neck may cause urinary incontinence or require a more complex repair i.e. ureteral reimplantation, in the setting of an injury to the ureteral orifice.

**Table 1:** American Association for the Surgery of Trauma Bladder Organ Injury Scale.

Bladder Injury Description		
Grade	Injury	Description
I	Hematoma	Contusion, intramural hematoma
	Laceration	Partial thickness
II	Laceration	Extraperitoneal bladder wall laceration <2 cm
III	Laceration	Extraperitoneal ≥ 2 cm or intraperitoneal <2 cm bladder wall laceration
IV	Laceration	Intraperitoneal bladder wall laceration ≥ 2 cm
V	Laceration	Laceration extending into bladder neck or ureteral orifice (trigone)

**Clinical Presentation**

Prompt recognition of bladder trauma can prevent severe complications due to urinary leakage, which include sepsis, peritonitis, abscess, urinoma, fistulas, and electrolyte disturbances through reabsorption.^12^ Morbidity and mortality from bladder injuries have been shown to correlate with injury severity scores >15, systolic blood pressure <90 mmHg, and concomitant pelvic fractures.^2^ Bladder injuries are also associated with longer hospital stays and carry a significant risk of morbidity and potential for increased cost of care.^9^

Gross hematuria, seen in 67-95% of cases, is the most classical symptom associated with bladder trauma.^14,15^ Microscopic hematuria may be seen in 5% of cases.^16^ Other signs such as the mechanism of injury, associated pelvic fracture, suprapubic tenderness, low urine output, difficulty voiding, elevated creatinine, abdominal hematoma, edema of the perineum and upper thighs, and shock should all raise the index of suspicion for a bladder injury.^17,18^ In the case of penetrating injuries, especially GSWs, entrance and exit wounds in the lower abdomen, perineum and buttocks may be visualized and should be traced.^13^

Iatrogenic bladder injuries during surgery may present with clear fluid or appearance of the urethral catheter in the surgical field, blood or gas in the urine drainage bag, fatty tissue or bowel seen on cystography, low return of bladder irrigation fluid, and inability to distend the bladder or conversely abdominal distension.^8^ This should prompt urological consultation. ^19^

While isolated bladder injuries are infrequent, risk factors include young age, male gender, alcohol intoxication, and trauma.^20^ Alcohol causes distension of the bladder and increases the risk of blunt trauma from motor-vehicle accidents. Isolated bladder injuries may have a delay in presentation and diagnosis, sometimes of up to five days, resulting in increased blood urea nitrogen and creatinine through reabsorption in the peritoneum.^20^ Therefore, a high index of suspicion in the emergency room should be maintained for patients presenting with risk factors mentioned previously.

**Clinical Assessment**

Trauma patients should undergo assessment per Advanced Trauma Life Support protocol developed by the American College of Surgeons. Hemodynamically unstable patients should not undergo acute evaluation of bladder trauma, but rather be taken for immediate surgical exploration.^21^ Gross hematuria in the setting of a pelvic fracture is an absolute indication for cystography, as bladder injury is present in 29% of such cases.^16,20^ Gross hematuria refers to visible blood from the urinary tract while microscopic hematuria can only be detected on urinalysis. Gross hematuria without pelvic fracture and microscopic hematuria with pelvic fractures are relative indications for cystography if there is clinical suspicion. Clinical suspicion may include mechanism of injury, pubic symphysis diastasis, >1 cm obturator ring fracture displacement, penetrating injuries with pelvic trajectories, inability to void, low urine output, increased blood urea nitrogen or creatinine, abdominal distension, suprapubic pain, or urinary ascites seen on imaging. A small number of patients with pelvic fractures (0.6-5%) will present with microscopic hematuria; however, microscopic hematuria, in general, is a poor predictor of bladder injury.^16,22,23^ In a study by Brewer et al., of 214 patients who underwent cystography for microscopic hematuria, none were found to have a bladder injury.^24^ Thus, cystography for the presence of a pelvic fracture or microscopic hematuria alone is not recommended.^25^

While X-ray cystography has been traditionally used to evaluate for bladder injury, most centers are moving towards the utility of Computed Tomography (CT) cystography due to increased convenience and rapid turnover time.^9^ CT cystography is particularly beneficial when other abdominal organs require imaging, as it can detect multiple injuries including the source of hematuria. The European Association of Urology (EAU) recommends CT cystography be used in the context of other possible abdominal trauma, while the American Urological Association (AUA) guidelines do not specifically address the use of CT versus X-ray. 

For both CT and X-ray cystography, contrast is instilled in the bladder in a retrograde manner via gravity filling through a catheter. The bladder is commonly distended with at least 300 mL of contrast material. X-ray cystography requires a minimum plain film, complete filling film, and post-drainage film. The post-drainage film is utilized to identify a posterior bladder injury that may be masked by a bladder filled with contrast. Oblique X-ray images may also be used to help delineate the location of a bladder injury. In comparison, the post-drainage film is not required in CT cystography since three-dimensional reconstruction allows for circumferential evaluation of the bladder and localization of the laceration.^26^

CT cystography is equally effective as retrograde cystography in the diagnosis of bladder rupture with similar specificity and sensitivities.^24,25,27^ Furthermore, one study showed that the findings of CT cystography matched findings after operative exploration of bladder trauma in 82% of cases, and had a sensitivity and specificity of detecting bladder rupture of 95% and 100%, respectively.^28^ Compared to X-ray cystography, CT is more expensive and confers greater radiation. However, CT takes less time and includes more detail of the surrounding pelvic structures. While both are equally effective in detecting bladder rupture, we expect the trend to continue towards CT cystography. 

**Figure 1:**
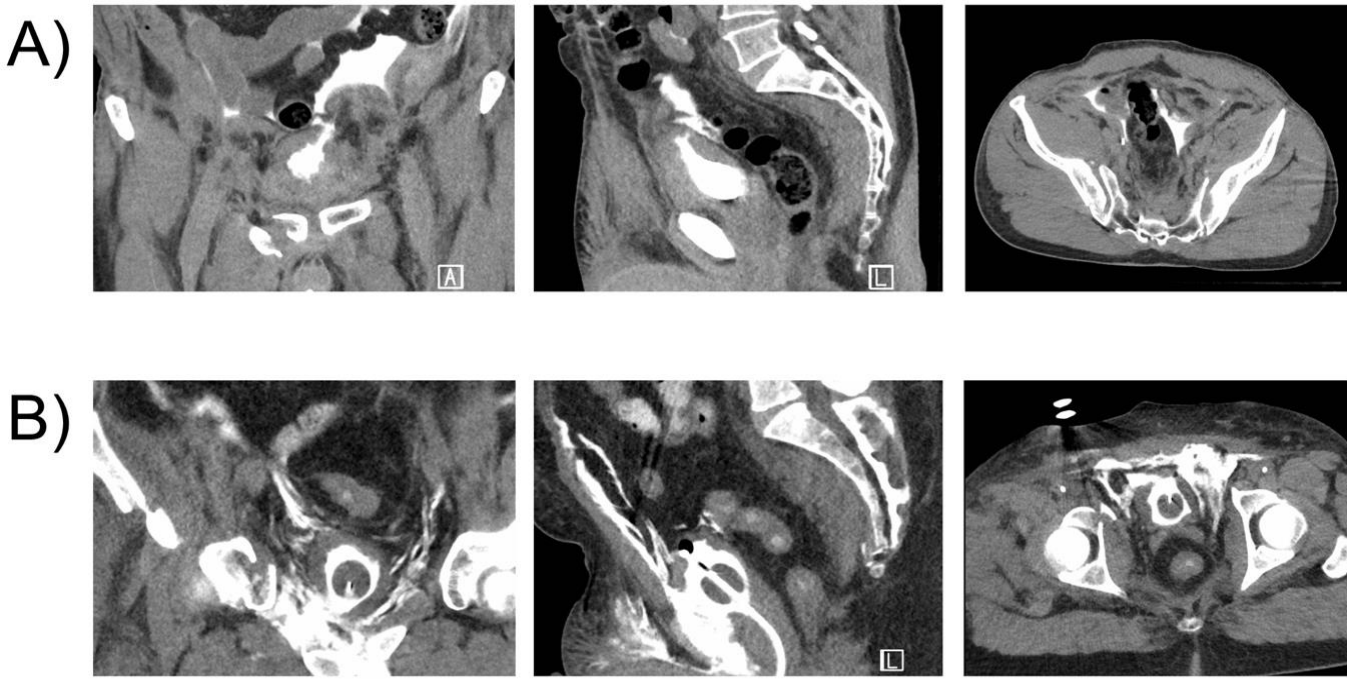
Coronal, sagittal, and axial images for an A) intraperitoneal and B) extraperitoneal bladder injury.

Contrast material outside the bladder is an indication of bladder injury (Figure 1). In IP ruptures, contrast material may extravasate into the paracolic gutters and outline loops of bowel. In EP ruptures, contrast material is seen in the retropubic space, anterior peritoneal spaces, and between the superficial soft tissue layers of the thighs.^29^ Conversely, in the case of bladder contusion or interstitial bladder injury, there is no contrast extravasation outside the bladder. Contusions appear normal on cystography while interstitial injuries may present as an intramural hematoma.^11^

In the case of an intra-operative bladder injury, the EAU guidelines recommend the use of cystoscopy for evaluation of suspected bladder injuries. Alternatively, for patients undergoing intraabdominal surgery, an indwelling urethral catheter may be filled while the abdomen is inspected for fluid extravasation from the bladder. While routine cystoscopy after gynecological or urological procedures is controversial, it is warranted if bladder injury is suspected after hysterectomies, sling operations (especially via retropubic route), or transvaginal mesh procedures.^13^ This is important as bladder injuries may be missed. In one study 67% of bladder injuries during hysterectomy were not detected until after cystoscopy.^30^

**Management**

A bladder contusion is a diagnosis of exclusion in patients presenting with hematuria in the setting of blunt trauma for which no observable cause is found. Contusions do not necessitate treatment unless significant hemorrhage is present for which a large bore catheter can be used for drainage and irrigation if required.^11^ Interstitial bladder injuries can be managed with prolonged bladder rest with a urethral catheter, and a repeat cystogram is not necessary.^11^

Surgical management of a bladder injury is warranted for IP injuries since they carry the risk of sepsis, tend to be larger injuries, and have a higher associated risk of morbidity and mortality when compared to EP injuries.^12^ IP injuries therefore require surgical exploration, which is usually performed through a lower midline or Pfannenstiel incision. The laceration should be sutured in one or two layers with an absorbable running suture. After the bladder injury has been repaired, the closure may be tested by filling the bladder in a retrograde fashion through a urethral catheter. Furthermore, use of a colored agent, such as methylene blue, may help to identify leaks during bladder filling. An abdominal drain may also be placed to evaluate for post-operative urine leaks. There are no current guidelines on the optimal length of time for catheter placement after bladder repair, but 7-14 days has been reported and is commonly used^3^ AUA guidelines recommend against using suprapubic catheters following bladder repairs, as urethral catheters are sufficient in the majority of cases. ^22^ In fact, drainage with urethral catheters have been associated with shorter hospital stays and lower morbidity compared to combined drainage with suprapubic and urethral catheters.^31^

EP injuries are usually managed conservatively, with bladder drainage via catheter followed by a cystogram to confirm healing of the injury. In a study by Johnsen et al., cystogram revealed continued extravasation in at least 18% of patients with EP injuries managed with catheters, suggesting confirmatory cystography may still be of some utility.^32^ The majority of ruptures heal by three weeks; if the injury has not healed by four weeks, AUA guidelines recommend surgical repairs.^12^ The guidelines also recommend surgery for EP bladder injuries when there is persistent hematuria, associated pelvic organ injury, the presence of foreign bodies or projecting bones in the bladder, ongoing urinary leak, and penetrating trauma.^22^ Other indications may include concomitant vaginal or rectal lacerations, inadequate drainage via urethral catheters, bladder neck injuries, and internal fixation of pelvic fractures.^33^ Concurrent cystorrhaphy during surgical intervention for other abdominal injuries has also been shown to reduce urologic complications, time in intensive care, and overall hospital stay.^34^ Similarly, EAU guidelines recommend concomitant cystorrhaphy during laparotomy to decrease infective complications.^13^

## Conclusion

Bladder injuries while uncommon, carry a significant risk of morbidity and mortality if not detected and treated promptly. Gross hematuria is a classical sign of bladder injury. Other signs and symptoms include microscopic hematuria, suprapubic tenderness, hematomas, and low urine output. CT and X-ray cystography are equally effective in detecting the location and type of bladder injuries. Most bladder injuries can be managed conservatively with urethral catheter drainage, but surgery is indicated with intraperitoneal injuries and some specific instances of extraperitoneal injuries. While this review focused exclusively on bladder injuries, it is important to remember that ureteral or urethral injuries may co-exist with bladder injury. Therefore, the entirety and extent of the genitourinary tract should be considered when determining management.
